# Associations between vitamin A and K intake and lung function in the general US population: evidence from NHANES 2007–2012

**DOI:** 10.3389/fnut.2024.1417489

**Published:** 2024-09-20

**Authors:** Yi-Chuan Chen, Ming-Szu Hung

**Affiliations:** ^1^Department of Emergency Medicine, Madou Sin-Lau Hospital, The Presbyterian Church in Taiwan, Tainan, Taiwan; ^2^Department of Pulmonary and Critical Care Medicine, Chang Gung Memorial Hospital, Chiayi, Taiwan; ^3^Department of Respiratory Care, Chang Gung University of Science and Technology, Chiayi, Taiwan; ^4^School of Medicine, Chang Gung University, Taoyuan, Taiwan

**Keywords:** lung function, vitamin A, vitamin K, airway obstruction, forced

## Abstract

**Introduction:**

While nutrition's critical role in enhancing respiratory health is acknowledged, the specific impacts of vitamins A and K on lung function remain largely unexplored. The study aimed to evaluate the relationships between vitamins A and K intake and lung function.

**Methods:**

The cross-sectional study focused on adults aged 20–79 with utilizing data from US National Health and Nutrition Examination Survey (NHANES) 2007–2012. Lung function was assessed by measuring forced expiratory volume (FEV1), forced vital capacity (FVC), and the ratio of these two values (FEV1/FVC). Regression model was performed to determine the associations between intake of vitamins A and K and outcomes.

**Results:**

Data of 10,034 participants (representing 142,965,892 adults in the US) were analyzed. After adjusting for relevant confounders, multivariable analysis revealed 1 μg/day increase of vitamin A intake was significantly associated with 0.03 ml increased FEV1 (*p* = 0.004) and 0.04 ml increased forced vital capacity (FVC) (*p* < 0.001). In addition, 1 μg/day increase in vitamin K intake was significantly associated with 0.11 ml increased FEV1 (*p* = 0.022). Neither vitamin A and K intake was associated with FEV1/FVC or presence of airway obstruction.

**Conclusions:**

In relatively healthy population of the US, greater vitamin A or K intake was independently associated with better lung function assessed by spirometry. Benefits of such vitamins for pulmonary health should be confirmed in future randomized controlled trials.

## Introduction

The ability of the lungs to intake and expel air efficiently is critical in maintaining the oxygen levels of the body and ensuring the removal of carbon dioxide. Lung function, a vital indicator of respiratory health, can be influenced by various factors, including diet (1), environmental exposure (2), genetic predisposition (3), and aging (4).

Disorders that negatively impact lung capacity and airflow include a wide range of conditions, such as asthma (5) and pulmonary fibrosis (6), both of which significantly disrupt breathing mechanics. These conditions vary significantly in their underlying pathophysiology and implications for patient health.

Moreover, as individuals age, lung function naturally declines due to various physiological changes. These changes include decreased lung elasticity, reduced respiratory muscle strength, and structural alterations in the alveoli (7). In normal aging, these factors contribute to a gradual reduction in lung capacity and airflow, affecting physical endurance and increasing susceptibility to respiratory illnesses.

There is a growing interest in understanding how dietary micronutrients, especially vitamins, impact lung function in both healthy individuals and those with lung diseases. For instance, vitamin D has long been associated with lung function in both healthy individuals and patients with lung diseases (8, 9). Primarily obtained from dark green vegetables, vitamin K is considered to play a role in the physiology of the airway and lung alveolar structure (10). In a recent study, lower levels of vitamin K were associated with reduced ventilatory capacity and a higher risk of asthma, wheezing, and chronic obstructive pulmonary disease (COPD) (11).

Similarly, vitamin A, another fat-soluble micronutrient, is also essential for the formation of lung alveoli. Chronic vitamin A deficiency can lead to decreased alveolar septation and significant changes in the respiratory epithelium, potentially resulting in lung functional defects and related diseases. A previous review has linked vitamin A deficiency with impairments in lung physiology and the development of pulmonary diseases (12). However, the evidence regarding the relationships between vitamin A and K consumption and lung function in the healthy general population is insufficient, and it has yet to be evaluated from a population-based perspective.

Understanding the potential benefit of dietary nutrients on lung function may provide healthcare professionals with an evidence-based lifestyle strategy to better counsel patients toward improved pulmonary health. Therefore, we conducted this study to evaluate the associations between vitamin A and K intake and lung function parameters in the general adult population of the US using the data obtained from a nationally representative database.

## Materials and methods

### Data source

This population-based, cross-sectional study analyzed the data obtained from the National Health and Nutrition Examination Survey (NHANES) database, which is managed by the Centers for Disease Control and Prevention (CDC), specifically through the National Center for Health Statistics (NCHS) in the US (http://www.cdc.gov/nchs/nhanes/).

The NHANES is designed to evaluate the health and nutritional status of adults and children across the USA. It utilizes a complex, multistage design to collect and analyze data that accurately represent the national, non-institutionalized population of the USA. The data are made available for research purposes, and the permission to use the data is granted to researchers by the NCHS.

Participants in NHANES first complete a household interview and are then invited to undergo an extensive examination at a mobile examination center (MEC), including a physical examination, specialized measurements, and laboratory tests. Consequently, the data collected through NHANES are both reliable and multidimensional and can be equated to a population-level assessment (13).

### Ethics statement

This study was approved by the Institutional Review Board (IRB) of Chang Gung Memorial Hospital (IRB number 202401041B1). The NCHS Research Ethics Review Board reviewed and approved the NHANES, and all survey participants provided written informed consent. Therefore, no further informed consent was required. For further details, please refer to the NHANES website for NCHS Research Ethics Review Board Approval (https://www.cdc.gov/nchs/nhanes/irba98.htm). Additionally, all NHANES data released by the NCHS are de-identified and remain anonymous during data analysis.

### Study population selection

The present study used data from the US NHANES database for the 2007–2012 cycles. Adults aged 20–79 years were eligible for inclusion. The exclusion criteria were as follows: no complete data on forced expiratory volume in 1 s (FEV1) or forced vital capacity (FVC), airway obstruction status, vitamin K or A intake, body mass index (BMI), education level, smoking status, and a history of malignancy, asthma, or physician-diagnosed chronic bronchitis or emphysema. These conditions were identified through the questionnaire of 'Medical conditions' from the NHANES, which asked participants, “Has a doctor or other health professional ever told you that you had asthma/cancer/chronic bronchitis/emphysema?”.

### Study variables

#### Assessment of daily intake of vitamins A and K

Data regarding the participants' food intake over 2 non-consecutive days were collected through 24-h dietary recall interviews as part of the NHANES survey. Dietary nutrient intake was estimated using the USDA's Food and Nutrient Database for Dietary Studies (FNDDS). The Recommended Dietary Allowance (RDA) for vitamin A in adults is set at 900 μg RAE/day for men and 700 μg RAE/day for women (14). Due to the lack of sufficient data to estimate an average requirement of vitamin K, an adequate intake (AI) is set based on representative dietary intake data from healthy individuals, in which the AI for men and women is 120 and 90 μg/day, respectively (14, 35).

#### Assessment of lung function and airway obstruction

Spirometry maneuvers were conducted using Ohio 822/827 dry-rolling seal volume spirometers used in the study were manufactured by Ohio Medical, located in Gurnee, Illinois, USA, to assess lung function. Participants who reported chest pain or physical problems, such as difficulty exhaling forcefully, receiving oxygen therapy, undergoing recent surgery, or having recent episodes of coughing up blood, were excluded from the study. Additionally, individuals with a history of retinal detachment or lung collapse were also excluded.

The spirometry measurements were evaluated according to the American Thoracic Society (ATS) data quality standards. Only those measurements that met or exceeded these standards were included in the analysis. Specifically, the criteria required either (A) three acceptable curves and two reproducible curves with two observations within 100 mL or (B) three acceptable curves and two reproducible curves with two observations within 150 mL (15).

The study outcomes were FVC, FEV1, and their ratio (FEV1/FVC), as measured using prebronchodilator spirometry. Based on previous studies, participants with FEV1/FVC <0.70 or the lower limit of normal were considered to have airway obstruction (16, 17).

#### Covariates

Demographic data, including age, gender, poverty-income ratio, race, and education level, were obtained by trained enumerators using household and sample demographic questionnaires, along with the Computer-Assisted Individual Investigation (CAPI) system (Confirmit Corp., New York, US). The collected data were weighted according to the NHANES protocol to ensure representativeness.

BMI values were calculated from measurements taken during the NHANES examination, defined as weight in kilograms divided by height in square meters. Body weight was measured using an electronic load cell scale, and standing height was measured using a stationary stadiometer.

Hypertension was identified in participants who responded “yes” to the questions, “Were you told on two or more different visits that you had hypertension, also called high blood pressure?” and “Because of your (high blood pressure/hypertension), have you ever been told to… take prescribed medicine?”.

Additionally, hypertension was defined by an average of three consecutive systolic blood pressure readings of ≥140 mmHg or an average of three consecutive diastolic blood pressure readings of ≥90 mmHg.

Participants were identified as having diabetes if they met any of the following criteria: a positive response to the questions, “Are you taking insulin?,” “Did a doctor tell you that you have diabetes?,” or “Do you take pills to lower blood sugar?” laboratory measurements showing an HbA1c of ≥6.5%, a fasting glucose level of ≥126 mg/dL, or a glucose level of ≥200 mg/dL in the oral glucose tolerance test (OGTT) (18).

Cardiovascular disease (CVD), including coronary heart disease, angina, congestive heart failure, myocardial infarction, and stroke, was identified through the question, “Has a doctor or other health professional ever told you that you have the disease?”.

The glomerular filtration rate (GFR) was estimated using re-calibrated serum creatinine with the 4-variable Modification of Diet in Renal Disease (MDRD) Study equation. Specifically, we used the IDMS-traceable MDRD Study equation, which uses standardized creatinine levels: GFR = 175 × (standardized serum creatinine)^−1.154^ × (age)^−0.203^ × 0.742 (if the participant is a woman) × 1.212 (if the participant is Black). Estimated GFR is reported in mL/min/1.73 m^2^. Chronic kidney disease (CKD) was defined as an eGFR of <60 mL/min/1.73 m^2^.

Smoking status was categorized into three groups: non-smoker (having smoked fewer than 100 cigarettes in a lifetime), former smoker (having smoked more than 100 cigarettes in a lifetime but not currently smoking), and current smoker (having smoked more than 100 cigarettes in a lifetime and currently smoking, based on a “yes” response to the question “Do you smoke now?”.

Excessive alcohol consumption was defined for participants who reported drinking alcohol ≥4 times/week in response to the question, “In the past 12 months, how often did you drink any type of alcoholic beverage?”.

Sedentary time was assessed by the NHANES through questions about daily hours spent watching TV, playing video games, or using a computer. The responses were categorized into three categories: <3, 3–6, and ≥6 h per day.

The total energy intake (kcal/day) was estimated from a dietary interview conducted by the NHANES that assessed the types and amounts of foods and beverages consumed by participants in the 24 h before the interview.

### Statistical analysis

The NHANES uses a complex, multistage, probability sampling design to ensure national representation, wherein sampling weights (WTDRD1), pseudo-stratum (SDMVSTRA), and pseudo-cluster (SDMVPSU) provided by the NHANES were applied in all analyses, following the guidelines set by the NCHS. In NHANES, the weights are developed to correct for the complex survey design (including oversampling), non-participation in the survey, and post-stratification adjustments to match total population counts from the Census Bureau. A specific sample weight is assigned to each person in the sample. This weight is a measure of the number of people in the population represented by that sample person. Food intake can vary by day of the week, so the use of MEC weights would disproportionately represent intakes on weekends. Hence, we selected WTDRD1 as the weight of the study, which is used in the analysis of Day 1 dietary recall data (either alone or when Day 1 nutrient data are used in conjunction with MEC data). Details about the sample weight can be found on the NHANE website: https://wwwn.cdc.gov/nchs/nhanes/tutorials/Weighting.aspx.

Categorical data were analyzed using the PROC SURVEYFREQ procedure and are presented as unweighted counts with weighted percentages. Continuous data were analyzed using the PROC SURVEYREG procedure and are presented as means with standard errors (SEs).

To determine the associations between lung function parameters, airway obstruction, and the study variables, the linear regression analysis was performed using SURVEYREG and SURVEYLOGISTIC procedures. Variables that showed significance in the univariate analysis were included and adjusted in the multivariable models. A two-sided *p*-value of <0.05 was considered statistically significant. All statistical analyses were performed using SAS statistical software (version 9.4, SAS Inc., Cary, NC, USA). All data were analyzed in 2023.

## Results

### Study population

**Figure 1:**
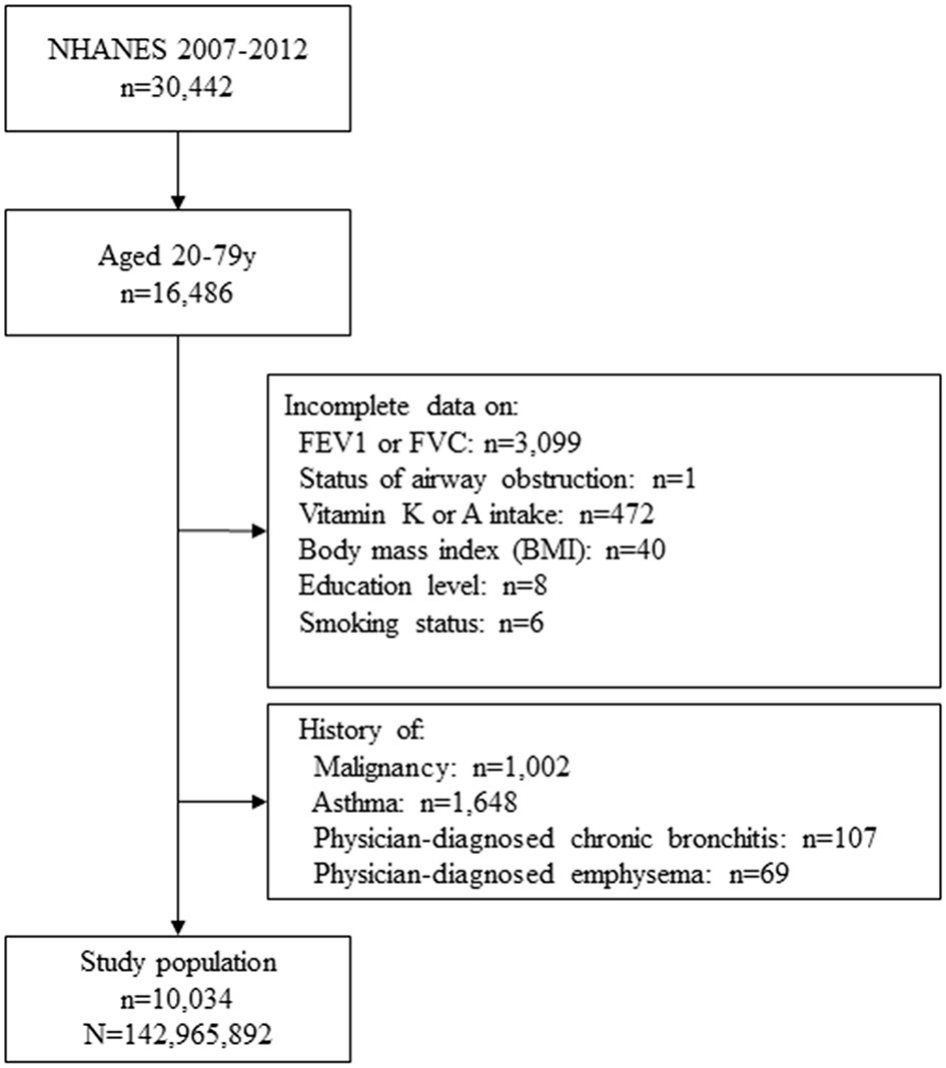
Flow diagram of study cohort selection.

Figure 1 depicts the flow chart of the study cohort selection process. A total of 30,442 participants were identified in the NHANES 2007–2012 cycles. Among these participants, 16,486 were aged between 20 and 79 years. After excluding subjects with incomplete data on FEV1, FVC, airway obstruction status, vitamin A or K intake, BMI, education level, smoking status, and those with a history of malignancy, asthma, physician-diagnosed chronic bronchitis or emphysema, a total of 10,034 subjects were included in the final analyses. Based on the sample weights provided by the NHANES, this cohort represents a population of 142,965,892 adults in the entire US after weighting (Figure 1).

### Characteristics of the study cohort

The mean age of the cohort was 43.9 years. The majority of them were Non-Hispanic White (66.1%), were not poor (85.2%), and had never smoked (never smokers; 56.1%). The mean FEV1 and FVC among all the participants were 3,295.0 and 4,179.8 mL, respectively. The mean daily intake of vitamin A was 653.5 μg/d, and for vitamin K, it was 112.3 μg/d. Approximately three-quarters (72.5%) of the participants consumed vitamin A below the RDA, and 70.3% of participants consumed vitamin K below the AI level. Hypertension was one of the most common comorbidities, affecting 26.6% of the study population (Table 1).

**Table 1:** Characteristics of the study population.

**Study variables**	**Overall**
	***n*** = **10,034**
**Lung function**	
FEV1, mL	3,295.0 ± 14.4
FVC, mL	4,179.8 ± 16.8
FEV1/FVC	0.8 ± 0.002
Airway obstruction	1,264 (12.8)
**Vitamin A**, μ**g/day**	653.5 ± 16.1
Above RDA	2,383 (27.5)
Below RDA	7,651 (72.5)
**Vitamin K**, μ**g/day**	112.3 ± 3.3
Above AI	2,693 (29.7)
Below AI	7,341 (70.3)
**Age, years**	43.9 ± 0.4
20–29	1,942 (21.5)
30–39	2,000 (20.3)
40–49	1,947 (21.6)
50–59	1,741 (19.6)
60–69	1,579 (11.7)
70–79	825 (5.3)
**Gender**	
Men	5,141 (51.0)
Women	4,893 (49.0)
**Race**	
Non-Hispanic White	4,034 (66.1)
Non-Hispanic Black	2,168 (11.4)
Hispanic	1,126 (6.0)
Others	2,706 (16.5)
**BMI, kg/m** ^2^	28.5 ± 0.1
Normal (18.5–24.9)	2,823 (30.1)
Underweight (<18.5)	137 (1.5)
Overweight (25–29.9)	3,442 (34.3)
Obese (≥30)	3,632 (34.1)
**Poverty income ratio**	
Not poor: >1	7,245 (85.2)
Poor: ≤ 1	1,952 (14.8)
Missing	837
**Education level**	
High school and above	7,440 (83.2)
Never attended high school	2,594 (16.8)
**Smoking status**	
Never	5,700 (56.1)
Former	2,132 (22.0)
Current	2,202 (21.8)
**Total energy intake, kcal/day**	2,216.1 ± 13.9
**DM**	1,263 (8.5)
**Hypertension**	3,145 (26.6)
**CVD**	565 (4.3)
**CKD**	554 (5.0)
**Excessive alcohol consumption**	679 (8.2)

AI, adequate intake; CVD, cardiovascular disease; CKD, chronic kidney disease; DM, diabetes mellitus; RDA, Recommended Dietary Allowance; FEV1, forced expiratory volume in the first second; FVC, forced vital capacity.

### Associations between vitamin A intake, FEV1, FVC, FEV1/FVC, and airway obstruction

The results of the multivariable analysis exploring the associations between lung function and vitamin A and K intake are summarized in Tables 2, 3. After adjusting for relevant confounders, 1 μg/day increase in vitamin A intake was significantly associated with a 0.03 mL increase in FEV1 (*p* = 0.006) and a 0.04 mL (*p* < 0.001) increase in FVC. In addition, individuals with vitamin A intake above the RDA had significantly higher FEV1 (adjusted β = 40.22, *p* = 0.046) and FVC (adjusted β = 60.43, *p* = 0.018) compared to those with intake below the RDA. However, no significant associations were found between vitamin A intake and the FEV1/FVC ratio or airway obstruction (Tables 2, 3).

**Table 2:** Associations between FEV1, FVC, vitamin A, and K intake.

	**FEV1, mL** ^**a**^		**FVC, mL** ^**b**^	
	**Adjusted** β	***P*** **-value**	**Adjusted** β	***P*** **-value**
	**(95% CI)**		**(95% CI)**	
**Vitamin A**, **μg/day**	0.03 (0.01, 0.05)	**0.006**	0.04 (0.02, 0.06)	**<0.001**
Below RDA	Ref		Ref	
Above RDA	40.22 (0.75–79.69)	**0.046**	60.43 (10.93, 109.93)	**0.018**
**Vitamin K**, **μg/day**	0.11 (0.02, 0.20)	**0.022**	0.12 (−0.02, 0.26)	0.082
Below AI	Ref		Ref	
Above AI	17.07 (−18.12, 52.27)	0.334	0.79 (−48.47, 50.04)	0.975

^a^Adjusted for age in years, gender, race, BMI (continuous), education level, smoking status, total energy intake, DM, hypertension, CVD, and CKD.

^b^Adjusted for age in years, gender, race, BMI (continuous), poverty income ratio, education level, smoking status, total energy intake, DM, hypertension, CVD, CKD, and excessive alcohol consumption.

AI, adequate intake; CVD, cardiovascular disease; CKD, chronic kidney disease; DM, diabetes mellitus; RDA, Recommended Dietary Allowance; FEV1, forced expiratory volume in the first second; FVC, forced vital capacity. Bold values indicate statistically significant results (*p* < 0.05).

**Table 3:** Associations between FEV1/FVC, airway obstruction, vitamin A, and K intake.

	**FEV1/FVC (%)** ^**a**^		**Airway obstruction** ^**b**^	
	**Adjusted** β	***P*** **-value**	**Adjusted OR (95% CI)**	***P*** **-value**
**Vitamin A**, **μg/day**	−0.000004 (−0.0002, 0.0001)	0.950	1.00 (1.00, 1.00)	0.155
Below RDA	Ref		Ref	
Above RDA	−0.09 (−0.50, 0.31)	0.648	1.10 (0.88, 1.38)	0.412
**Vitamin K**, **μg/day**	−0.0002 (−0.001, 0.0008)	0.659	1.00 (0.999, 1.001)	0.845
Below AI	Ref		Ref	
Above AI	−0.03 (−0.45, 0.40)	0.89	1.05 (0.87, 1.26)	0.595

^a^Adjusted for age in years, gender, race, poverty income ratio, education level, smoking status, total energy intake, DM, hypertension, CVD, CKD, and excessive alcohol consumption.

^b^Adjusted for age in years, gender, race, BMI (continuous), education level, smoking status, DM, hypertension, CVD, and excessive alcohol consumption.

AI, adequate intake; CVD, cardiovascular disease; CKD, chronic kidney disease; DM, diabetes mellitus; RDA, Recommended Dietary Allowance; FEV1, forced expiratory volume in the first second; FVC, forced vital capacity; OR, odds ratio; CI, confidence interval.

### Associations between vitamin K intake, FEV1, FVC, FEV1/FVC, and airway obstruction

After adjusting for relevant confounders, 1 μg/day increase in vitamin K intake was associated with a 0.11 mL increase in FEV1 (*p* = 0.022). However, no significant associations were found between vitamin K intake and FVC, the FEV1/FVC ratio, or airway obstruction (Tables 2, 3).

## Discussion

The results of our study demonstrate that, among a relatively healthy US population who had never been diagnosed with chronic respiratory diseases (i.e., asthma, chronic bronchitis, or emphysema), higher dietary intakes of vitamins A and K are associated with improvements in specific lung functional parameters.

In particular, higher dietary intake of vitamin A is independently correlated with better FEV1 and FVC, while higher intake of vitamin K is independently correlated with better FEV1.

These findings suggest that vitamins A and K may offer potential benefits for lung health, although causal inferences could not be made due to the cross-sectional design of this study. This study suggests that increasing dietary intake of vitamins A and K may improve lung health in healthy individuals, emphasizing the need for further prospective and interventional studies to confirm these findings and potentially inform nutritional guidelines.

Maintaining lung function is essential for a healthy life (19). To prevent chronic respiratory conditions, smoking cessation remains the most important strategy (20). Additionally, numerous studies have shown that a proper diet rich in antioxidants can help prevent the development of COPD and mitigate its progression (21–24).

In a study focusing on antioxidants, Shen et al. found that consuming recommended levels of vitamin K or vitamin A significantly reduced the likelihood of emphysema, suggesting that these vitamins may be crucial for maintaining lung health (25). Specifically, this study documented that individuals who consumed the recommended levels of vitamins K and A were 39 and 33% less likely to have emphysema, respectively.

However, there are three major differences between the study conducted by Shen et al. and the present one. First, the present study focused on a relatively healthy population, excluding individuals diagnosed with asthma, chronic bronchitis, or emphysema. Second, Shen et al. relied on self-reported questionnaires to identify emphysema without using objective spirometry data, limiting the interpretability of their findings.

The role of Vitamin K in the human body extends beyond its well-known function in blood coagulation. It is considered to affect airway physiology (26). The relationship found between vitamin K and lung function may be mediated through the pathway of elastin degradation (27).

As proposed in previous studies, vitamin K can activate protein S for anticoagulation and prevent the production of inflammatory cytokines involved in the cytokine storm seen in acute lung injury (28). In addition, the matrix Gla protein (MGP), a potent inhibitor of arterial calcification, is also widely expressed in the lungs (29).

Vitamin K is also essential for the carboxylation of MGP, and insufficient levels of vitamin K can hinder MGP activation, potentially leading to lung damage in conditions such as SARS-CoV-2 pneumonia and other lung diseases, including COPD (30).

On the other hand, vitamin A, along with its analogs and derivatives, forms the retinoid family, which is involved in various physiological processes, including vision, immune responses, cellular differentiation and proliferation, and embryonic development (31). Recent research has also identified new biological functions for vitamin A, including roles in insulin resistance, lipid metabolism, energy balance, and redox signaling (32).

Vitamin A deficiency negatively impacts lung health by promoting elastin degradation and oxidative injury (12). An animal study showed that vitamin A deficiency during growth is associated with changes in the extracellular matrix and increased levels of TGF-β in the lung tissue (33).

Furthermore, vitamin A deficiency causes an imbalance between the production of reactive oxygen species and the antioxidant defenses in the lungs (34). This imbalance may partly explain the association found between higher vitamin A consumption and improved lung function parameters in our study.

The implications of this study are significant for both clinical practice and public health nutrition. The findings suggest that higher dietary intakes of vitamins A and K are associated with improved lung function parameters in a relatively healthy US population without chronic respiratory diseases, indicating that these micronutrients may offer potential benefits for maintaining and possibly enhancing lung health.

These results highlight the importance of ensuring adequate dietary intake of vitamins A and K and suggest that healthcare professionals should consider incorporating nutritional counseling into their strategies for promoting respiratory health. However, while these associations are promising, they necessitate further prospective and interventional studies to establish causality and inform future dietary guidelines and public health policies aimed at preventing respiratory conditions through optimal nutrition.

## Strengths and limitations

A significant strength of our study is the use of data from the NHANES, which are both comprehensive and nationally representative, enhancing the generalizability of our findings to the overall US population. Furthermore, our analysis included data of objectively measured, standardized lung function parameters, with adjustments made for various confounding factors, making the results reliable and robust.

However, there are certain limitations to be considered. Since this is a cross-sectional study, we could not determine a causal relationship between vitamins A and K and lung function. The assessment of vitamin A and K intake was based on 24-h dietary recall interviews, which may have introduced bias due to potential inaccuracies in reporting. Moreover, physical activity, which might affect spirometry results, was not included in the analysis because of a high number of missing values. The study also did not account for whether participants lived in urban or rural areas, a potential confounder that could affect lung function, as this information was not available. Finally, we were unable to assess changes in spirometry over time, limiting our ability to evaluate long-term effects.

## Conclusion

In conclusion, this study highlights the independent associations between higher intake of vitamins A and K and improved lung function in the general US adult population. However, further prospective studies or randomized controlled trials are needed to establish causal relationships and confirm the potential benefits of these vitamins in improving pulmonary health.

## Data Availability

The original contributions presented in the study are included in the article/supplementary material, further inquiries can be directed to the corresponding author.
